# Digital signature schemes with strong existential unforgeability

**DOI:** 10.12688/f1000research.72910.1

**Published:** 2021-09-16

**Authors:** Jason Chia, Ji-Jian Chin, Sook-Chin Yip

**Affiliations:** 1 Faculty of Engineering, Multimedia University, Cyberjaya, Selangor, 63100, Malaysia; 2 Faculty of Computing and Informatics, Multimedia University, Cyberjaya, Selangor, 63100, Malaysia

**Keywords:** Cryptography, Digital Signatures, Strong Existential Unforgeability

## Abstract

Digital signature schemes (DSS) are ubiquitously used for public authentication in the infrastructure of the internet, in addition to their use as a cryptographic tool to construct even more sophisticated schemes such as those that are identity-based. The security of DSS is analyzed through the existential unforgeability under chosen message attack (EUF-CMA) experiment which promises unforgeability of signatures on new messages even when the attacker has access to an arbitrary set of messages and their corresponding signatures. However, the EUF-CMA model does not account for attacks such as an attacker forging a different signature on an existing message, even though the attack could be devastating in the real world and constitutes a severe breach of the security system. Nonetheless, most of the DSS are not analyzed in this security model, which possibly makes them vulnerable to such an attack. In contrast, a better security notion known as strong EUF-CMA (sEUF-CMA) is designed to be resistant to such attacks. This review aims to identify DSS in the literature that are secure in the sEUF-CMA model. In addition, the article discusses the challenges and future directions of DSS. In our review, we consider the security of existing DSS that fit our criterion in the sEUF-CMA model; our criterion is simple as we only require the DSS to be at least secure against the minimum of existential forgery. Our findings are categorized into two classes: the direct and indirect classes of sEUF-CMA. The former is inherently sEUF-CMA without any modification while the latter requires some transformation. Our comprehensive  review contributes to the security and cryptographic research community by discussing the efficiency and security of DSS that are sEUF-CMA, which aids in selecting robust DSS in future design considerations.

## Introduction

**Figure 1.**
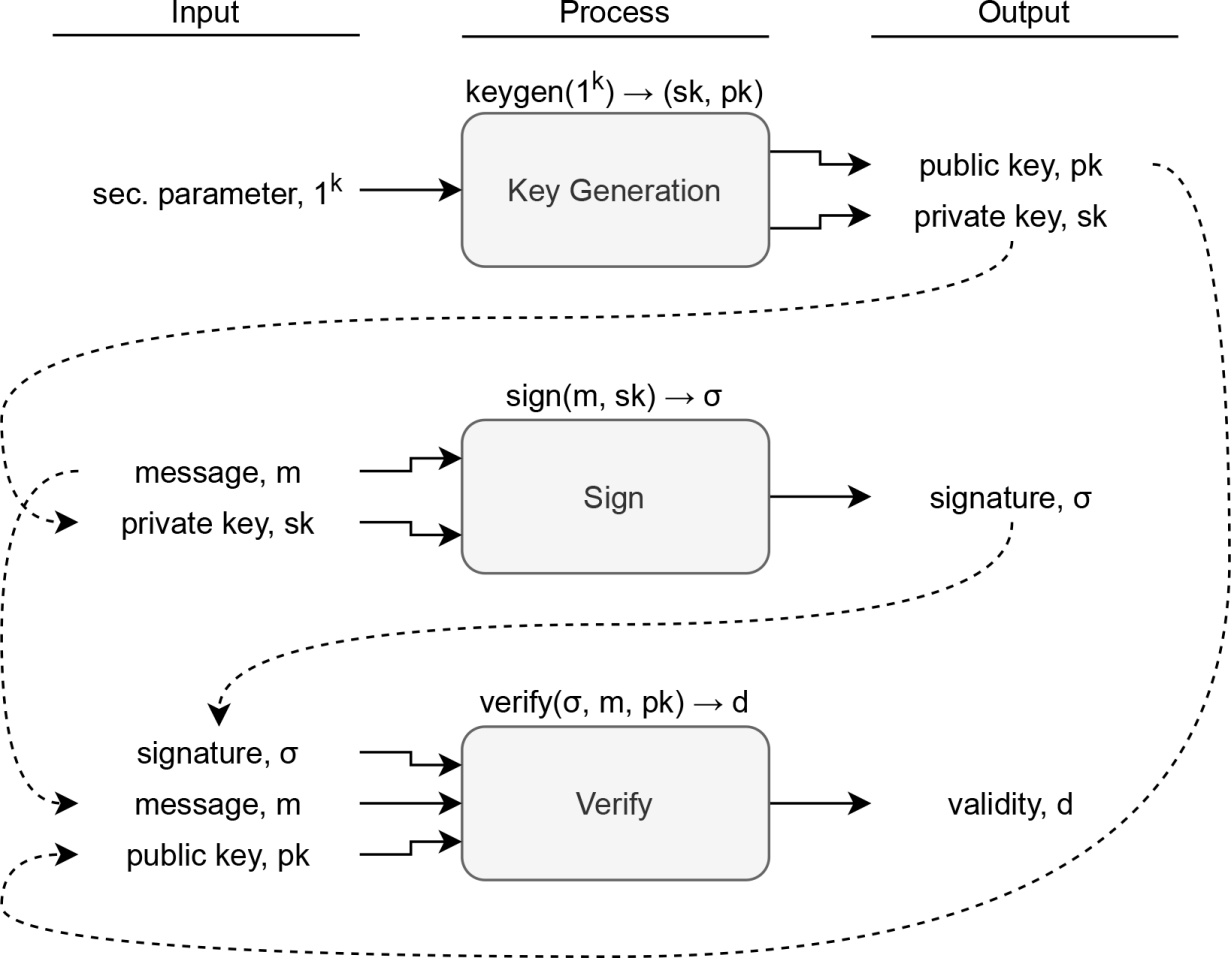
The three processes of a digital signature scheme and the relationship between the input/outputs.

The idea of a digital signature scheme (DSS) was proposed by Diffie and Hellman in 1976 as a necessity to design efficient authenticated electronic communications which can serve as legal evidence in the court of law. ^1^ Rivest, Shamir, and Adleman realized the idea in their seminal work known as the RSA cryptosystem, ^2^ the first of many. A DSS consists of three processes, as shown in Figure 1.

For every ( *sk*, *pk*) ← keygen(1 *k*) and every *m*, verify(sign( *m*, *sk*), *m*, *pk*) = 1 must hold. A *σ* on *m* is valid if verify( *σ*, *m*, *pk*) = 1. This is a standard (informal) definition of DSS. ^3^ In the early 90s, a paradigm known as hash-then-sign forms the industry standard for issuing digital signatures. ^4^ The idea is to sign on the hash of a message, h←H(m) instead of the *m* itself; this has a few benefits for *h* is constant size, which leads to efficient signing on speed and a constant sized signature. Verification would then require the verifiers to first validate the signed hash, then perform hashing $h^{\prime }\leftarrow H(m)$ before finally comparing *h*′ and *h.* Concrete examples of the hash-then-sign are discussed in. ^5- 15^

## Properties of DSS

The following properties are required by DSS ^3^:

• *Public verifiability*: A signature *σ* generated from a private key *sk* can be verified using a public key *pk.* This property differentiates DSS from other integrity protection mechanisms, such as message authentication codes (MAC). A consequential result from this property is that signatures are also *transferrable*, meaning a party can copy *σ* and *pk* to use it to convince others that the message is authentic from the signer.
• *Non repudiation*: A signer cannot later deny that they have authenticated a message *m* once the signature of *m*, *σ* is generated and known. This is also another property that separates DSS from MAC, because the only entity that could have plausibly generated the signature in the case of DSS must possess the private key *sk*; whereas in a MAC scheme, the keys are shared. This property also implies that only the one in posession of *sk* can generate valid signatures, which disallows forgeries.

### Message recovery

**Figure 2.**
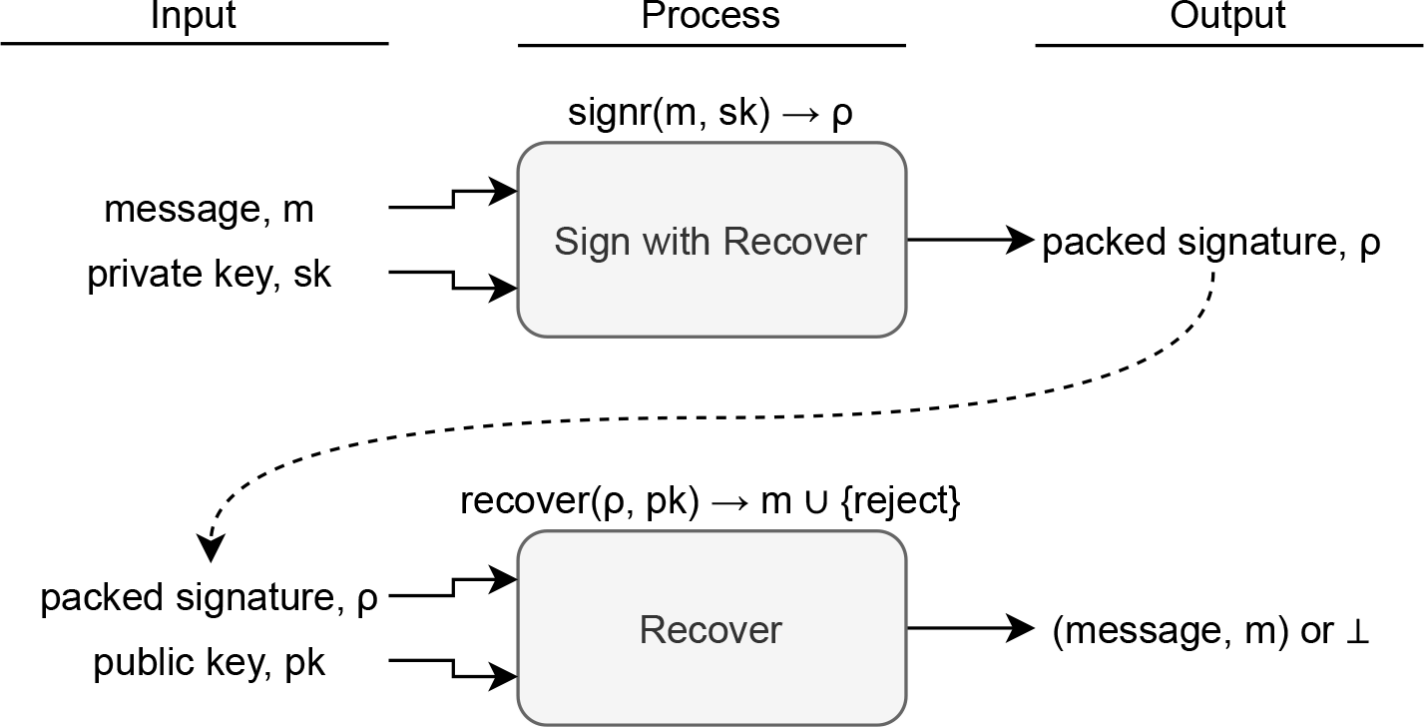
Sign and verify process for a digital signature scheme with message recovery.

Notice that the verification process requires both the message and the signature as inputs, requiring the signer to transmit both. Alternatively, some DSS can support *message recovery.* DSS with message recovery (DSS-R) has a different sign and verification process, shown in Figure 2; the signer only needs to transmit a packed signature *ρ*, and the verifier would recover the message *m* successfully or abort ⊥ depending on signature validity. For practical purposes, | *ρ*|≤| *σ*| + | *m*|. Examples of DSS-R are found in previous literature. ^16- 23^

### Security models

**Figure 3.**
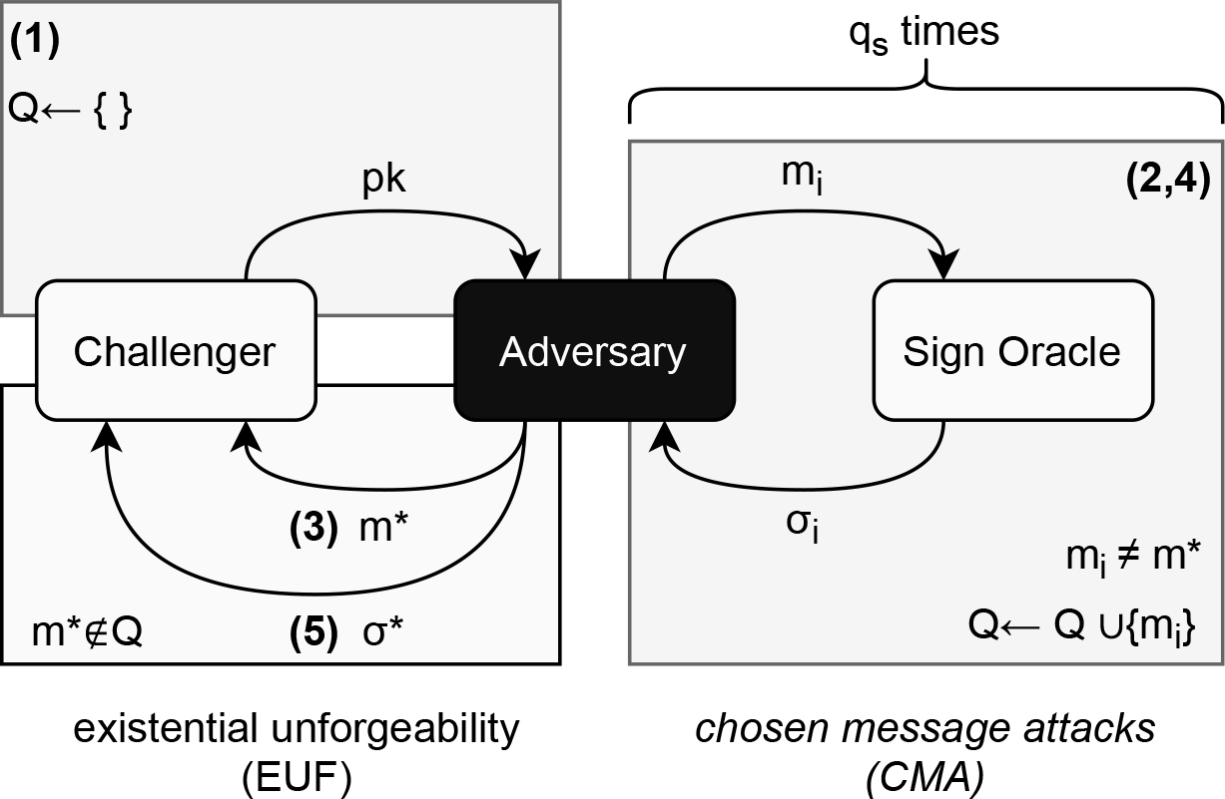
EUF-CMA security model.

The security of DSS was first formalized by Goldwasser, Micali and Rivest in 1988. ^24^ Different security goals are used to model the different security guarantees of a DSS when faced with an adversary. The commonly accepted goal to model against is the goal of *existential forgery* (EUF), which is the easiest for attackers to achieve among other goals (e.g., *selective forgery, total break*). In addition, ^24^ defined the adversarial capabilities for a DSS, which aims to model what types of attacks can be carried out by an adversary when attempting to break the DSS. The strongest capability, known as *adaptive chosen message* (CMA) is the widely accepted adversarial capability that is the most used in the literature on DSS. Figure 3 shows the interactions of a *challenger* and an adversary in the EUF-CMA model. In step (1), the challenger sets up an empty set *Q* and gives the *pk* to the adversary. In step (2), the adversary may make oracle queries that model its chosen message attack capability. The queried messages are added to the set *Q.* In step (3), the adversary announces to the challenger the target message m* it wants to forge. m* must not be an element of *Q*, nor it can be queried to the oracle; this prevents trivial attacks which uses the oracle to break the security goal. Note, step (4) allows the adversary to use the signing oracle again, which models the *adaptive* nature of the attack. Finally, in step (5), the adversary outputs a forgery σ*. We say the adversary breaks the DSS if σ* on m* is valid. *q _s_* quantifies the number message-signature pair made available to the adversary.

### Strong existential unforgeability (sEUF-CMA)

**Figure 4.**
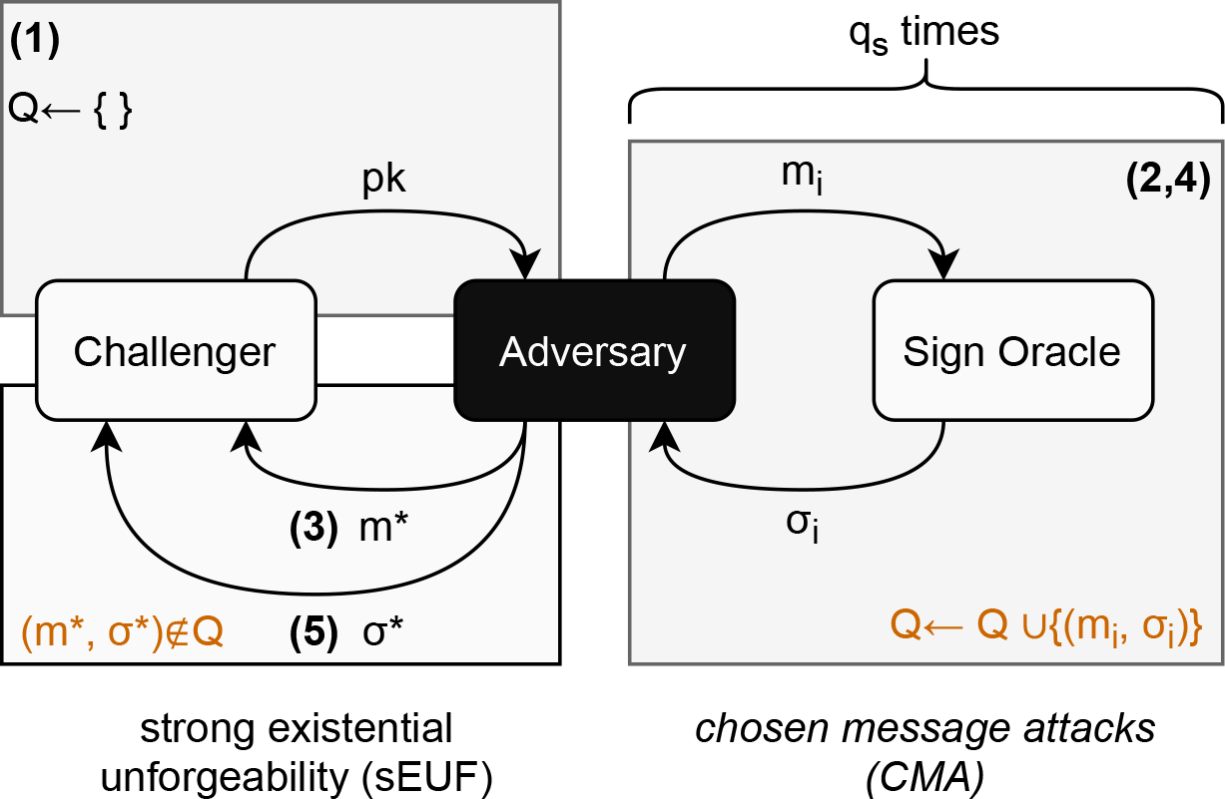
sEUF-CMA security model.

Consider the case for *randomized* signatures in the EUF-CMA model (e.g., DSA ^13^ or PSS-R ^18^). A randomized DSS allows multiple valid signatures for a single message, which has a subtle implication on the model: Suppose that the adversary queried for a message-signature pair ( *m,σ*). Now, the adversary forges a *different* valid signature *σ*′ ≠ *σ* on the same message *m.* This is an easier security goal, but could be a critical vulnerability when the DSS is used in a scenario in which the designers assumed that no *new* signatures can be forged, because a *different* signature on an existing message is still *new.* In other words, EUF-CMA does **not** guarantee that if that an attacker knows ( *m,σ*), it cannot forge ( *m,σ′*) such that *σ′* on *m* is valid. This gave rise to a stronger security model, known as strong existential unforgeability or sEUF-CMA. ^25^ Figure 4 shows the interactions of the adversary with the challenger in the sEUF-CMA model. The main difference is the constraint during the chosen message attacks and in the final step (5). Notably, the adversary can even query for signatures on the challenge message m*, but may not submit any of the signatures obtained from the sign oracle as forgeries. In contrast to EUF-CMA, sEUF-CMA ensures that an adversary cannot produce any new signatures at all; *any* valid signature must have originated from the signer.

## Why does sEUF-CMA matter?

Let’s take a step back and consider why sEUF-CMA is even worth considering in the first place. DSS is very often used as a building block to construct other cryptographic schemes. For example, using the Fiat-Shamir transform, ^26^ a DSS can be turned into an identification protocol. DSS has also been used to create identity-based identification schemes, ^27^ signcryption schemes, ^25^ authenticated key exchanges, ^28^ and identity-based encryption schemes. ^29^ DSS with EUF-CMA security is found to be insufficient in some of the constructions, particularly to build non-malleable cryptographic schemes. In a nutshell, non-malleability refers to the impossibility of an adversary to generate a *different* ciphertext to some previously known ciphertext that decrypts to the same message, which is a desirable property in cryptographic schemes. ^30^ Thus, if a DSS is sEUF-CMA, it is much more versatile because it can be useful as a building block for many of the schemes which requires the property of non-malleability.

### A simple attack for when a DSS is not sEUF-CMA

**Figure 5.**
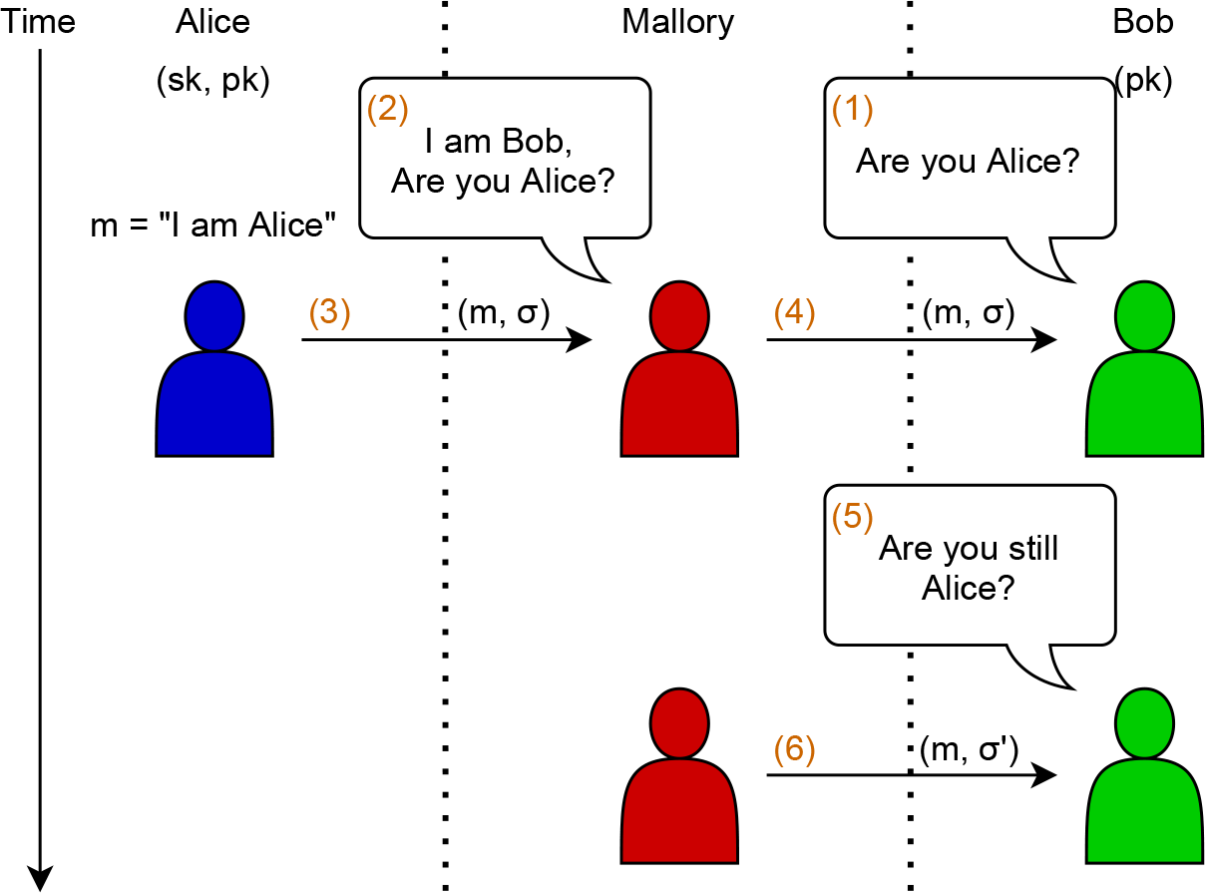
A simple problem on a digital signature schemes without sEUF-CMA.

We show a toy example of a simple attack that can be achieved by an attacker if the DSS used for authentication is not sEUF-CMA. Figure 5 shows honest users Alice and Bob, as well as an attacker Mallory which has hijacked the channel. In step (1), Bob wants to authenticate that Alice is truly on the other end. Mallory launches a chosen message attack in step (2) and (3) on Alice and obtains the message *m* and signature *σ.* In step (4), Mallory forwards ( *m,σ*) to Bob, which may initially convince them. After some time elapsed, Bob wants to re-authenticate Alice to ensure that they are still who they claim to be (5). Mallory forges a valid signature *σ*′ ≠ *σ* on *m* and forwards that to Bob (6). From Bob’s perspective, Alice generating a *different* signature ought to warrant some confidence that Alice is truly Alice. However, if the DSS is not sEUF, this is **not** the case, as Bob is obviously duped.

### Real life problems arising from signature malleability (non-sEUF)

We established sEUF-CMA is of theoretical interest. Here we briefly mention a few real-life needs for sEUF-CMA to emphasize the importance of it. Not too long ago, Stern *et al.* showed how to duplicate ECDSA signatures based on their malleability. ^31^ A more recent example is the transaction malleability of the popular cryptocurrency, Bitcoin. When a transaction in Bitcoin is signed, the signature does not cover the entire transaction used for hashing; an attacker could perform strong forgery (i.e., *maul* the signature), then claim that the transaction has failed (because the hash is not valid). The sender would believe it because the signature is valid! This leads to the sender issuing a new transaction, without knowing that the original transaction is valid. ^32, 33^ Decker and Wattenhofer pointed out that this subtle flaw could be responsible for a small portion of Bitcoins stolen during the 2014 MtGox attack. ^34^ They remarked that transaction malleability should be carefully considered when implementing Bitcoin clients. In another research, Jackson *et al.* found that signatures that are not sEUF-CMA may cause the *strong session agreement* of a well-known authenticated key exchange algorithm, the STS-ISO ^1^ to fail. ^36^ They analyzed STS-ISO using the Tamarin Prover and found that if sEUF is violated, attackers can force the parties in the session to accept message that do not originate from either of the honest parties.

## DSS secure in sEUF-CMA

The notion of sEUF-CMA first appeared in the form of non-malleability of ciphertexts in the works of Dolev, Dwork and Naor ^30^ in 1991. The term sEUF-CMA was first introduced by An, Dodis and Rabin which presented it as requirement to construct signcryption schemes. ^25^ Boneh, Shen and Waters were the first DSS that is considered and proven in the sEUF-CMA model, ^37^ but mentions that DSS that are sEUF-CMA have existed earlier such as full domain hash (FDH)-RSA. ^18^ FDH-RSA must be sEUF-CMA because the signatures generated are determined exactly by the message only. This means that FDH-RSA signatures are *deterministic.* For deterministic signatures, a strong forgery cannot occur without the message *m*′ being different from the original *m*, therefore, deterministic signatures are *generally* sEUF-CMA. We see that this is also the case for the widely used and popular EdDSA which had recently received a provable security treatment. ^38^ Boneh, Shen and Waters shows that *non-deterministic* signatures may also exhibit sEUF-CMA, such as the Micali-Reyzin signatures, ^39^ Goh-Jarecki signatures ^40^ and Boneh-Boyen signatures. ^11^ The reason for this is that if the forger manages to re-randomize a signature on the same message, the signature constitutes an existential forgery through clever binding of the messages. This is a recurring paradigm to design sEUF-CMA signature schemes. ^11, 41^ Since the introduction of the sEUF-CMA model, existing EUF-CMA secure DSS are now re-considered in the sEUF-CMA model. An example of such work is by Fersch, Kiltz and Poettering on the well established DSA. ^13^ DSA was shown to be secure under sEUF-CMA, while ECDSA was not ^2^. ^43^ More recently in 2021, Brendel *et al.* proved the IETF version of EdDSA to be sEUF-CMA secure. ^38^ Table 1 shows a list of DSS that are secure under sEUF-CMA.

**Table 1.** DSS that are directly sEUF-CMA.

Scheme	Req.	Sec. Assump	Sign Cost	Ver. Cost	Sig. len.	M.R.	Std.
Lamport ^44^	N/A	1-way functions	|1k| hash/0	|1k| hash	|m|×|hash(⋅)|	✗	✓
DSA ^13, 43^	random oracle	DLP	0/ex	ex	2| ${\mathbb{Z}}_{q}$ |	✗	✗
FDH-RSA ^18^	random oracle	RSA	0/ex	ex	| ${\mathbb{Z}}_{n}$ |	✗	✗
CS99 ^45^	N/A	Strong RSA	0/4ex	4ex	3| ${\mathbb{Z}}_{n}$ |	✗	✓
BLS01 ^9^	random oracle	GDH	0/ex	pair	|G|	✗	✗
BMS03 ^46^	N/A	CDH	ex( $\log_{2}n_{m}$ )/ex	(pair + ex)( $\log_{2}n_{m}$ +1)	$\left(\log_{2}n_{m}+1)|G\right|$	✗	✓
GJ03 ^47^	random oracle	DDH	0/2ex	2ex	2| ${\mathbb{Z}}_{q}$ |	✗	✗
KW03 ^47^	random oracle	RSA	0/ex	ex	| ${\mathbb{Z}}_{n}$ |	✗	✗
BB04 ^11^	N/A	Strong DH	0/ex	pair	$\left|G|+|{\mathbb{Z}}_{q}\right|$	✗	✓
mNR04 ^48, 49^	N/A	GGM	0/ex	2ex	2| ${\mathbb{Z}}_{q}$ |	✓	✓
GJK +07-1 ^50^	random oracle	CDH	0/3ex	2ex	— G — + 2| ${\mathbb{Z}}_{q}$ | + 1	✗	✗
GJK +07-2 ^50^	random oracle	DDH	2ex/0	2ex	2| ${\mathbb{Z}}_{q}$ |	✗	✗
TP09 ^51^	random oracle	RSA & DLP	0/2ex	3ex	2| ${\mathbb{Z}}_{n}$ |	✗	✗
AGH +11 ^52^	N/A	GGM	0/(2+kmn)ex	(kmn)pair	3|G|	✗	✓
EdDSA ^38, 53^	random oracle	ECDLP	0/2ex	2ex	2|G|	✗	✗
NTC19 ^54^	random oracle	1-way trapdoor sampleable relations	0/2ex	2ex	|G|+1	✗	✗

Req. - Requirements; Sec. Assump - Security Assumptions; Sign Cost is partitioned into off-line/on-line costs, separated by a slash /; Ver. Cost - Verification Cost; Sig. Len. - Signature Length; M.R. - Supports message recovery; Std. - Standard Model as oppose to random oracle model; DLP - Discrete Logarithm Problem; RSA - RSA problem; GDH - Gap Diffie-Hellman; CDH - Computational Diffie-Hellman; ECDLP - Elliptic Curve Discrete Logarithm Problem; GGM - Generic Group Model; DDH - Decisional Diffie-Hellman Problem; ex - Exponentiation (or equivalent to point multiplication in elliptic curves); pair - Pairing operation; hash - hash operation; G group element; ${\mathbb{Z}}_{q}$ integer of order *q* (a scalar in elliptic curves); $G_{x}$ X coordinate of elliptic curve point; *n _m_* total number of messages; *k _mn_* key matrix size (see ^52^); Costs ignore cheap operations such as integer multiplication/addition or elliptic curve point addition/doubling. Whenever possible, the Strauss-Shamir optimization is applied (see ^55^).

### Message recovery

From another perspective, we consider DSS with message recovery (DSS-R) candidates under sEUF-CMA. As pointed out by Ateniese and de Mediros, the modified Nyberg-Rueppel signature is sEUF-CMA secure. ^49^ While it is tempting to think that DSS-R must be sEUF-CMA, because modifying the signature will surely modify the message itself given that one can recover the message from the signature. However, we see that this is not the case as Ateniese and de Mediros found the original Nyberg-Rueppel signatures ^49^ to be insecure in sEUF-CMA.

### Conversions to sEUF-CMA

Boneh, Shen and Waters opened a new field in the research of DSS under sEUF-CMA: Interest in sEUF-CMA conversion starts to accumulate with various works being published. ^41, 56- 58^ Instead of working on individual DSS, the line of research focuses on creating *efficient* conversions to enhance existing generic DSS with EUF-CMA into sEUF-CMA. Some transforms do not even need EUF-CMA security at minimum, only requiring EUF-GMA, which is a weaker notion than EUF-CMA. ^59, 60^ Table 2 shows a list of conversion methods since 2006, including the use of DSS in leakage resilient settings. ^61- 64^

**Table 2.** Conversions that produces DSS with sEUF-CMA.

Scheme	DSS Req.	Add. Req.	Sec. Assump	Sign Cost	Ver. Cost	Sig. len.	Std.
MR00 ^39^	factoring-based Fiat-Shamir	random oracle	integer fact.	0/2ex	2ex	2| ${\mathbb{Z}}_{q}$ |	✗
BSW06 ^37^	EUF-CMA, *partitioned*	randomized trapdoor	CDH + trapdoor	0/sg + ex + td	vf + ex + td	|σ| + | ${\mathbb{Z}}_{q}$ |	✓
TOO06-1 ^56^	EUF-CMA	random oracle	DLP	sg + ex/0	vf + ex	|σ| + | ${\mathbb{Z}}_{q}$ |	✗
TOO06-2 ^56^	EUF-CMA	collision resistant hash	DLP + collision resist	sg + ex/0	vf + ex	|σ| + 2| ${\mathbb{Z}}_{q}$ |	✓
SPW07 ^57^	EUF-CMA	*strong* randomized trapdoor	trapdoor	0/sg + 2td	vf + 2td	|σ| + | ${\mathbb{Z}}_{q}$ |	✓
BS07 ^41^	EUF-CMA	2-tier DSS	EUF-CMA of 2-tier DSS	0/2sg + kg	2vf	2|σ| + |pk|	✓
Goldreich ^65^	EUF-CMA	1-time DSS	*strong* 1-time EUF DSS	sg( $\log_{2}n_{m}$ )/sg	vf $\left(\log_{2}n_{m}+1\right)$	$\left(\log_{2}n_{m})(|\sigma |+|pk|\right)$	✓
HWZ07 ^58^	EUF-CMA	1-time DSS	*strong* 1-time EUF DSS	sg + kg/sg	2vf	2|σ| + |pk|	✓
LKZ + 08-S ^59^	EUF-GMA, *deterministic*	N/A	N/A	sg + kg/sg	2vf	2|σ| + |pk|	✓
LKZ + 08-P ^59^	EUF-GMA, *deterministic*	N/A	N/A	sg/sg	2vf	2|σ| + |1k|	✓
LAS + 10 ^60^	EUF-GMA/CMA	GTOW chameleon hash	one-wayness of GTOW hash	sg/td	vf + td	|σ| + | ${\mathbb{Z}}_{q}$ |	✓
**DSS with leakage resilience under sEUF-CMA**							
KV09-2.1 ^61^	Lamport DSS		1-time use 1-way functions	|1k| hash/0	|1k| hash	|m|×|hash(⋅)|	✓
WT14 ^62^	EUF-FLR	*strong* randomized trapdoor	trapdoor leakage resilience	0/sig + 2td	vf + 2td	|σ| + | ${\mathbb{Z}}_{q}$ |	✓
WT15 ^63^	EUF-FLR	*strong* randomized trapdoor	trapdoor leakage resilience	0/sig + 2td + pair + ex	vf + 2td	$\left|\sigma |+|{\mathbb{Z}}_{q}|+|G|+|\pi \right|$	✓
HHP16 ^64^	EUF-FLR	1-time EUF-FLR DSS	*strong* 1-time EUF-FLR DSS	sg + kg/sig	2vf	2|σ| + |pk|	✓

DSS Req. - Requirement for DSS before using the conversion; Add. Req. - Additional requirements; EUF-GMA - Existential Unforgeability under Generic Chosen Message Attack; GTOW - Given Target One-Wayness (See ^60^); EUF-FLR - Existential Unforgeability with Full Leakage Resilience; sg - signature generation cost; td - trapdoor operation cost; kg - key generation cost; 1 ^*k*^ - security parameter; vf - signature verification cost; *σ* - underlying signature length; *pk* underlying signature public key length; *π* - Groth-Sahai proof statement (See ^66^).

### Widely used DSS that have malleable signatures

Through our research, we investigated some of the most used DSS in the industry on their security in the sEUF-CMA model. The following are the popular DSS that are **not** sEUF-CMA secure.

1. RSA PKCS#1.5 ^67^
2. ECDSA ^31, 43^
3. Ed25519 (Original, not IETF RFC 8032) ^38^

## Challenges and future direction

In recent work, there has been several post-quantum cryptographic DSS that incorporates the sEUF-CMA model during design. ^68- 71^ DSS in various other contexts (e.g., privacy preserving computation, multiparty computation) such as a homomorphic DSS, ^72^ group DSS, ^73^ and proxy DSS ^74- 76^ are being considered in sEUF-CMA as well. In addition, DSS in even more complex cryptographic settings such as in certificateless and identity-based settings are also using sEUF-CMA as their standard model for security. ^77- 80^ We see that the security goal post has been moved from EUF-CMA to sEUF-CMA in the span of a decade and believe this is the right direction forward as DSS is increasingly used in intricate security protocols, which cannot tolerate any design flaw that arises from as simple as malleable signatures.

## Conclusion

In this work, we provided a comprehensive review on what is strong unforgeability in DSS, why is it needed, which of the DSS are secure under the model and how to obtain it if the DSS is only existentially unforgeable. We surveyed and analyzed existing DSS in literature which are secure under sEUF-CMA, and noted the requirements, computational and storage efficiency as well as the security assumptions of each DSS to provide an overview of DSS under the much more secure model.

## Data availability

No data is associated with this article.

## Footnotes

^1^
STS stands for *Station-to-Station* and is an authenticated key exchange protocol due to Diffie *et al*. ^35^

^2^
Brown proved ECDSA to secure under sEUF-CMA, ^42^ but this was refuted through a series of trivial attacks posed by. ^43^
