# Supplementary material for: Digital signature schemes with strong existential unforgeability
Source: F1000Res. 2021 Sep 16;10:931. [Version 1] doi: 10.12688/f1000research.72910.1 (PMC9925878; doi:10.12688/f1000research.72910.1)
Supplement: Peer review history

# Peer review: Reviewer response for version 1

**DOI:** 10.5256/f1000research.76522.r162696

**Published:** 2023-02-13

**Authors:** Pin Shen Teh

**Affiliations:** 1 Manchester Metropolitan University, Manchester, UK

- The research objective was clear.
- The concept of the work was straightforward.
- The presentation style of the article was clear.
- Grammar mistakes are noticeable. The sentence structure and choice of words used can be more concise and precise, some terms used were somewhat inconsistent, making reading and understanding the article quite challenging.
- Further elaboration of why the three popular DSS that are not sEUF-CMA secure would be helpful.
- Overall the depth of the work is sufficient for the calibre of this journal.

Is the review written in accessible language?

Yes

Are all factual statements correct and adequately supported by citations?

Yes

Are the conclusions drawn appropriate in the context of the current research literature?

Yes

Is the topic of the review discussed comprehensively in the context of the current literature?

Yes

Reviewer Expertise:

Biometrics, Security, Machine Learning, Mobile Authentication

I confirm that I have read this submission and believe that I have an appropriate level of expertise to confirm that it is of an acceptable scientific standard.

# Peer review: Reviewer response for version 1

**DOI:** 10.5256/f1000research.76522.r97082

**Published:** 2021-11-03

**Authors:** Denis Chee-Keong Wong

**Affiliations:** 1 Lee Kong Chian Faculty of Engineering and Science, Universiti Tunku Abdul Rahman, Kampar, Malaysia

The paper performs a review on existing DSS together with its properties, such as public verifiability, non-repudiation, and ability for message recovering. Furthermore, a detailed discussion on DSS security models are included, which are the EUF-CMA and sEUF-CMA. The differences between both security models are elaborated upon in detail. Finally, the authors provided comprehensive information on existing DSS in Table 1 for comparison purposes. As a survey paper, this work is comprehensive and suitable to be indexed. The authors need to re-check some of the entries in Table 1 to ensure the information stated is correct - please look at the "GJK +07-1", and please ensure the group G used is the same for all schemes with |G| in the column of "sig. len.".

Is the review written in accessible language?

Yes

Are all factual statements correct and adequately supported by citations?

Yes

Are the conclusions drawn appropriate in the context of the current research literature?

Yes

Is the topic of the review discussed comprehensively in the context of the current literature?

Yes

Reviewer Expertise:

Algebraic cryptography, algebra

I confirm that I have read this submission and believe that I have an appropriate level of expertise to confirm that it is of an acceptable scientific standard.
